# Chicago Medical Society

**Published:** 1872-01-15

**Authors:** Wm. E. Quine

**Affiliations:** Secretary


### CHICAGO MEDICAL SOCIETY.

Office of Dr. N. S. Davis,

Jan. 15th, 1872.

Dr. Paoli in the chair.

Dr. N. S. Davis exhibited a fine specimen of perforated intestine which was taken from a patient who had died in Mercy Hospital. On admission the patient stated that three weeks before, having contracted gonorrhœa, he commenced taking Copaiba, but at the end of a week stopped it and took a powerful cathartic. He has not felt well since though he has been up more or less every day. The night before his admission he took three pills, and has vomited almost incessantly and had violent diarrhœa ever since. Examination:—Considerable emaciation; capillary circulation sluggish; extremities cold and blue; lips thin and retracted; tongue dry and coated; pulse small and feeble; abdomen distended and tense; sudamina; frequent vomiting of stercoraceous matter; intellect clear. Diagnosis:—Judging from the condition of the tongue, circulation, intestines, the nature of the matter vomited and the suddenness of collapse, the Doctor expressed the opinion to his class that the patient was laboring under typhoid fever and that perforation had occurred. The patient died 12 hours after admission. Autopsy:—Peritoneum inflamed; its surface covered with patches of lymph; its cavity containing a small quantity of bloody serum and feculent matter. Two perforations of the ilium just above the ilio-cæcal valve in the midst of ulcerated glands.

Dr. Paoli reported a case of well marked cancer of the uterus, in which he prescribed cundurango with the effect of impairing the appetite and causing nausea and obstinate constipation. He was obliged to abandon its use and to rely exclusively on tonics, anodynes and disinfecting washes.

Dr. Davis alluded to two cases of cancer in which he had used cundurango with negative results. And said further, that though it was generally believed that with our present resources we are incapable of retarding the progress of the disease, he felt certain that he had seen several cases in which no progress had been made after treatment was commenced. Two of these he reported in detail. The first, a poor washerwoman, appeared at Dr. Williard Parker's clinic, in New York, nearly thirty years ago, with well marked scirrhus in the stage of ulceration of both breasts. No encouragement was given to her by the surgeon. On returning sorrowfully to her home she encountered Dr. Davis, and arrangements were at once made to commence treatment. He prescribed Bi-chloride of Mercury and Conium internally, and Stramonium Ointment locally. The patient improved steadily, and finally became able to perform her usual labor. On removing from New York he lost sight of her at this stage of improvement. The second patient has been under his care quite recently. She consulted him as to the propriety of operation—which for the time was discountenanced. He never advises operation before the disease has been checked by medication. He accordingly prescribed Conium and Arseniate of Soda, and the morbid growths have slowly and gradually disappeared. The Doctor withholds entirely animal food in treating Cancer, and continues medication after removal by operation for at least a year.

Dr. Flood, a visitor from Hyde Park, reported a case of Erysipelas which was promptly cured by Tincture of the Chloride of Iron, in small doses, and poultices of flax seed. In a general discussion which ensued, Dr. Davis remarked that he had as much faith in the power of medicines to shorten an attack of Erysipelas as of vaccination as a preventive of small-pox. He does not believe, however, that we have any specific for the disorder, and he has frequently found it necessary to prescribe such measures as are required in the treatment of other kinds of inflammation.

Dr. Marguerat reiterated these views.

Dr. Blanchard was appointed essayist for the next meeting.

On motion the Society adjourned.

Wm. E. Quine, *Secretary*.

